# De novo transcriptome analysis and glucosinolate profiling in watercress (*Nasturtium officinale* R. Br.)

**DOI:** 10.1186/s12864-017-3792-5

**Published:** 2017-05-23

**Authors:** Jin Jeon, Sun Ju Bong, Jong Seok Park, Young-Kyu Park, Mariadhas Valan Arasu, Naif Abdullah Al-Dhabi, Sang Un Park

**Affiliations:** 1 Department of Crop Science, Chungnam National University, 99 Daehak-ro, Yuseong-gu, Daejeon, 34134 Korea; 2 Department of Horticulture, Chungnam National University, 99 Daehak-ro, Yuseong-gu, Daejeon, 34134 Korea; 3 LAS Inc., 16 Arayuk-ro, Gimpo City, 10136 Korea; 4 Department of Botany and Microbiology, Addiriyah Chair for Environmental Studies, College of Science, King Saud University, P. O. Box 2455, Riyadh, 11451 Saudi Arabia

**Keywords:** *Nasturtium officinale*, Watercress, Transcriptome, Glucosinolates

## Abstract

**Background:**

Watercress (*Nasturtium officinale* R. Br.) is an aquatic herb species that is a rich source of secondary metabolites such as glucosinolates. Among these glucosinolates, watercress contains high amounts of gluconasturtiin (2-phenethyl glucosinolate) and its hydrolysis product, 2-phennethyl isothiocyanate, which plays a role in suppressing tumor growth. However, the use of *N. officinale* as a source of herbal medicines is currently limited due to insufficient genomic and physiological information.

**Results:**

To acquire precise information on glucosinolate biosynthesis in *N. officinale*, we performed a comprehensive analysis of the transcriptome and metabolome of different organs of *N. officinale*. Transcriptome analysis of *N. officinale* seedlings yielded 69,570,892 raw reads. These reads were assembled into 69,635 transcripts, 64,876 of which were annotated to transcripts in public databases. On the basis of the functional annotation of *N. officinale*, we identified 33 candidate genes encoding enzymes related to glucosinolate biosynthetic pathways and analyzed the expression of these genes in the leaves, stems, roots, flowers, and seeds of *N. officinale*. The expression of *NoMYB28* and *NoMYB29*, the main regulators of aliphatic glucosinolate biosynthesis, was highest in the stems, whereas the key regulators of indolic glucosinolate biosynthesis, such as *NoDof1.1*, *NoMYB34, NoMYB51,* and *NoMYB122*, were strongly expressed in the roots. Most glucosinolate biosynthetic genes were highly expressed in the flowers. HPLC analysis enabled us to detect eight glucosinolates in the different organs of *N. officinale*. Among these glucosinolates, the level of gluconasturtiin was considerably higher than any other glucosinolate in individual organs, and the amount of total glucosinolates was highest in the flower.

**Conclusions:**

This study has enhanced our understanding of functional genomics of *N. officinale*, including the glucosinolate biosynthetic pathways of this plant. Ultimately, our data will be helpful for further research on watercress bio-engineering and better strategies for exploiting its anti-carcinogenic properties.

**Electronic supplementary material:**

The online version of this article (doi:10.1186/s12864-017-3792-5) contains supplementary material, which is available to authorized users.

## Background

*Nasturtium officinale* R. Br. is an aquatic perennial herb that generally grows in around clear, cold water. It is primarily found in Europe, North and South America, and Asia, where it is commonly known as “watercress.” In some regions, *N. officinale* is considered an aquatic weed and is consumed as a fresh salad plant or soup garnish, or used in other recipes [1, 2]. It is well documented that *N. officinale* is recognized as a valuable traditional medicinal plant, because of its numerous health-benefiting constituents, such as vitamins B, C, and E, pro-vitamin A, folic acid, carotenoids, glucosinolates, and many minerals, including Ca, Fe, I, and S [3, 4]. In particular, watercress contains high amounts of gluconasturtiin (2-phenethyl glucosinolate), which is hydrolyzed by myrosinase to produce 2-phennethyl isothiocyanate [5, 6]. This latter metabolite has been demonstrated to suppress carcinogen activation through the inhibition of phase I enzymes and induction of phase II enzymes [7]. Recent study has shown that watercress accessions from the University of South-ampton germplasm collection contain various gluconasturtiin contents and antioxidant (AO) capacity [8]. In addition, *N. officinale* is now known to play a role in the prevention of several other diseases including diabetes, inflammatory diseases [9], and lymphocyte DNA damage [10].

Glucosinolates are sulfur-rich anionic secondary metabolites derived from glucose and amino acids. Approximately 200 different glucosinolates are known to occur naturally in plants [11, 12] and are found almost exclusively within the order Brassicales. These compounds play roles in defense against pests and have various biological activities related to human health [13–17]. Glucosinolates can be classified into three main groups, depending on the content of modified amino acids: aliphatic glucosinolates, derived from methionine, isoleucine, leucine, or valine; aromatic glucosinolates, derived from tyrosine or phenylalanine; and indole glucosinolates, derived from tryptophan [18]. Biosynthesis occurs in three independent phases: (i) side chain elongation of precursor amino acids with an additional methylene group, (ii) partial amino acid conversion to form the core structure, and (iii) secondary modification of the amino acid side chain [19]. Several glucosinolate biosynthetic genes are generally involved in these three independent phases of glucosinolate biosynthesis. Elongation of methionine is initiated by *METHYLTHIOALKYLMALATE SYNTHASE* (*MAM*), *BILE ACID TRANSPORTER5* (*BAT5*), and *BRANCHED-CHAIN AMINOTRANSFERASE* (*BCAT*) [20–23]. Core structure formation of glucosinolates is accomplished in five steps via oxidation by cytochrome P450 of CYP79 and CYP83, followed by C-S lyase, *S*-glucosyltransferase, and sulfotransferase [24–26]. Finally, secondary modification is mediated by several genes, including *GS-OX*, *GS-AOP*, *GS-OH*, *BZO1*, and *CYP81F2* [19]. Furthermore, various transcription factors are implicated in the regulation of glucosinolate biosynthesis. HIGH ALIPHATIC GLUCOSINOLATE1 (HAG1)/MYB28, HAG2/MYB76, and HAG3/MYB29 are the main regulators of aliphatic glucosinolate biosynthesis [27, 28], whereas HIGH INDOLIC GLUCOSINOLATE1 (HIG1)/MYB51, HIG2/MYB122, and ALTERED TRYPTOPHAN REGULATION1 (ATR1)/MYB34 regulate indolic glucosinolate biosynthesis. Among these regulators, AtMYB34 and AtMYB51 play major roles in indolic glucosinolate biosynthesis and AtMYB122 is presumed to play an accessory role in indolic glucosinolate biosynthesis [29]. IQD1, a nuclear-localized calmodulin-binding protein, controls the biosynthesis of aliphatic and indolic glucosinolates [30]. AtDof1.1 induces the transcription of *CYP83B1* and increases the levels of aliphatic and indolic glucosinolates [31].

Whole transcriptome sequencing technologies have been widely utilized as powerful tools for high-throughput genotyping because they are inexpensive, rapid, accurate, and reproducible [32, 33]. Among next-generation sequencing (NGS) technologies, the Illumina sequencing platform [34] has been successfully used for de novo transcriptome sequencing of numerous species, such as rice (*Oryza sativa*) [35], maize (*Zea mays*) [36], soybean (*Glycine max*) [37], sweet potato (*Ipomoea batatas*) [38], barley (*Hordeum vulgare)* [39], chickpea (*Cicer arietinum*) [40], tea plant (*Camellia sinensis*) [41], and Chinese bayberry *(Myrica rubra)* [42].

In this study, we used an Illumina NextSeq500 sequencer to analyze the transcriptome of *N. officinale* seedlings and generated 69,570,892 raw reads that were assembled into 69,635 transcripts. The *N. officinale* transcriptome showed highest species similarity and annotation ratio to *Arabidopsis thaliana*. From the transcriptome data, we identified several candidate genes that encode enzymes related to glucosinolate biosynthetic pathways. To validate the spatial distribution of glucosinolate-related genes, we analyzed the expression of glucosinolate biosynthesis genes and transcription factors in different organs of *N. officinale* using quantitative real-time RT-PCR. Metabolite profiling using HPLC-UV analysis identified eight different glucosinolates in the different organs of *N. officinale*, and the total glucosinolate contents were found to be highest in flowers. Among the eight identified glucosinolates, the level of gluconasturtiin was considerably higher than that of any other glucosinolate, irrespective of the organ. Taken together, the data obtained from this comprehensive transcriptomic and metabolomic profiling will provide an invaluable resource for a better understanding of glucosinolate biosynthetic pathways, as well as strategies for exploiting the anti-carcinogenic properties in *N. officinal.*

## Methods

### Plant material and growth conditions

*Nasturtium officinale* seeds were purchased from Asia Seeds Co., Ltd (Seoul, Korea) and grown under field conditions at the experimental greenhouse of Chungnam National University (Daejeon, Korea). Different organs were harvested from mature plants at approximately 2 months after sowing. The samples were immediately frozen in liquid nitrogen and then stored at -80 °C for RNA isolation or freeze-dried for subsequent analysis by high performance liquid chromatography (HPLC).

### Illumina sequencing of the transcriptome

Total RNA was isolated from frozen seedlings of *N. officinale* using the RNeasy Mini Kit (Qiagen, USA) and cleaned by ethanol precipitation. We removed rRNAs in total RNA using the ribo-zero rRNA removal kit (Epicentre, RZPL11016) and constructed a cDNA library for RNA sequencing using the TruSeq stranded total RNA sample prep kit-LT set A and B (Illumina, RS-122-2301 and 2302) according to the manufacturer’s protocols (Illumina, San Diego, CA, USA). The cDNA library was sequenced in 76 bp length paired-end (PE) reads in an Illumina NextSeq500 sequencer (Illumina Inc., San Diego, CA, USA) to produce 69,570,892 raw sequencing reads.

### De novo assembly and annotation of the watercress transcriptome

The quality-trimmed reads of watercress RNAs were assembled as contigs of the watercress transcriptome using the Trinity software package (http://trinityrnaseq.github.io/) [43]. The Trinity program combines the overlapping reads of a given length and quality into longer contig sequences without gaps. The characteristic properties, including N50 length, average length, maximum length, and minimum length of the assembled contigs were calculated using Transrate software (http://hibberdlab.com/transrate) [44]. We clustered the watercress transcriptome contigs based on sequence similarity using CD-HIT-EST software (http://weizhongli-lab.org/cd-hit) [45]. To infer the biological functions of watercress transcripts, we performed a homology search of the transcripts in the various public protein and nucleotide databases. A BLASTX search was performed using the National Center for Biotechnology Information (NCBI) (http://blast.ncbi.nlm.nih.gov) nr and Clusters of Orthologous Group (COG) (http://www.ncbi.nlm.nih.gov/COG) protein databases, BRAD (http://brassicadb.org/brad) *Brassica rapa* protein database, TAIR (TAIR10, http://www.arabidopsis.org) *Arabidopsis thaliana* protein database, and the EBI Swiss-Prot (UniProt) database. A BLASTN search was performed using the NCBI nucleotide database. The best scored hit from the BLASTX and BLASTN results passed the cutoff of e-value < 10^−5^ and was selected for annotation of query transcripts for each database search. Transcript lists and sequences are presented in Additional files 1 and 2. The functional category distributions of watercress transcripts in terms of Gene Ontology (GO) and COG were evaluated using the results of the homology search. COG functional category information attached to the hit COG proteins was used for determining COG functional category distribution, and GO information attached to the hit UniProt proteins was collected and re-analyzed using the WEGO tool (http://wego.genomics.org.cn) [46] in terms of the level for the three GO categories.

### Differentially expressed gene analysis

To quantify watercress transcript expressions, we aligned preprocessed quality-trimmed reads on the watercress transcript sequences and calculated the expression values with the aligned read counts for each transcript. Bowtie2 (http://bowtie-bio.sourceforge.net/bowtie2) software [47] was used to align the quality-trimmed reads on the transcript sequences, and eXpress (http://bio.math.berkeley.edu/eXpress) software [48] was used to evaluate gene expression, in terms of fragments per kilobase of exon per million mapped fragments (FPKM), from the aligned results. The FPKM method provides a comparison between genes within a sample or between samples by normalizing the amount of sequencing for samples and gene length bias during gene expression evaluation.

### Identification of candidate genes related to glucosinolate biosynthetic pathways

We searched for candidate genes involved in glucosinolate biosynthetic pathways using functional annotation data based on the orthologous gene names. In addition, the glucosinolate biosynthetic genes of *Arabidopsis* obtained from TAIR were used as queries to search for homologous sequences in the watercress transcriptome database. Following this, each sequence was confirmed by the BLAST program in the NCBI GenBank database.

### Quantitative real-time RT-PCR

For quantitative real-time RT-PCR, gene-specific primer sets were designed for each gene using the Primer3 website (http://frodo.wi.mit.edu/primer3/). Real-time RT-PCR was performed in a CFX96 real time system (BIO-RAD Laboratories, USA) using 2x Real-Time PCR Smart mix (BioFACT, Korea) under the following conditions: 95 °C for 15 min, followed by 40 cycles of 95 °C for 15 s, annealing for 15 s at 56 °C, and elongation for 20 s at 72 °C. PCR products were analyzed using Bio-Rad CFX Manager 2.0 software. Gene expression was normalized to that of the *UBC9* gene, used as a housekeeping gene. The results of the real-time RT-PCR assay were calculated as the mean of three different biological experiments using seeds and different plant organs (all leaves, stems, roots, and flowers) of individual plants. Real-time RT-PCR product sizes and primer sequences are shown in Additional file 3: Table S1.

### Extraction and HPLC analysis of glucosinolates

HPLC-UV analysis of glucosinolates was performed according to previously described methods with some modification [49, 50]. Glucosinolates were extracted with 70% (v/v) methanol from 100 mg lyophilized powder in a water bath at 70 °C for 5 min. After centrifugation at 12,000 × *g* for 10 min, the supernatant was loaded onto a mini-column packed with DEAE-Sephadex A-25 (Sigma-Aldrich Co., Ltd., St. Louis, MO, USA). After an overnight reaction at ambient temperature, the desulfo-glucosinolates were eluted with 1.5 mL of high-purity water and filtered through a 0.45 μm hydrophilic PTFE syringe filter (Ø, 13 mm; Advantec, Tokyo, Japan) in a vial. Desulfo-glucosinolates were quantified using a 1260 series HPLC system (Agilent Technologies, CA, USA) equipped with an Inertsil ODS-3 (C18) column 150 × 3.0 mm i.d., particle size 3 μm (GL Science, Tokyo, Japan). HPLC analysis was performed with a flow rate of 0.4 mL min^−1^ at a column temperature of 40 °C and a wavelength of 227 nm. The solvent systems employed were (A) water and (B) 100% acetonitrile. The gradient program used was as follows: 0 min, 0% solvent B; 0–2 min, 0% solvent B; 2–7 min, 10% solvent B; 7–16 min, 31% solvent B; 16–19 min, 31% solvent B; 19–21 min, 0% solvent B; 21–27 min, 0% solvent B (total 27 min). The individual glucosinolates were determined by their HPLC peak area ratios and response factors (ISO 9167-1, 1992) with reference to a desulfo-sinigrin external standard.

## Results

### Sequencing and de novo assembly of the *N. officinale* transcriptome

**Fig. 1:**
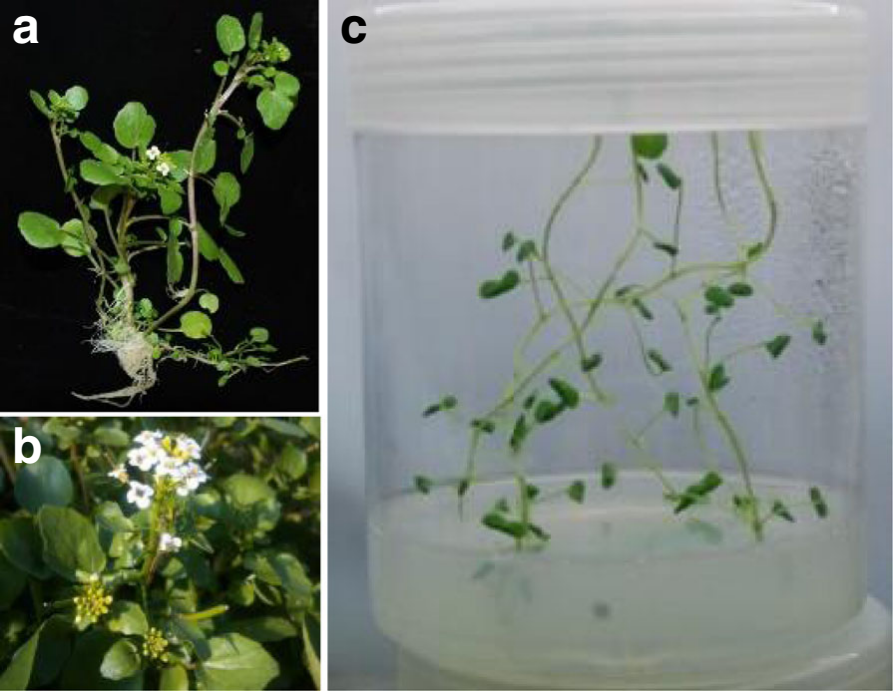
Photographs of mature plant (**a**), inflorescence (**b**), and seedling (**c**) of *N. officinale*

As shown in Fig. 1, watercress can grow to a height of 50 to 120 cm and has slender hollow stems and small round leaves (Fig. 1a). Tiny white flowers are formed in clusters and become small pods containing two rows of seeds (Fig. 1b). To provide an overview of the *N. officinale* transcriptome, we performed RNA-sequencing analysis of *N. officinale* seedlings using the Illumina NextSeq500 platform (Fig. 1c). After removal of adaptor sequences, 69,570,892 reads comprising a total of 5,287,387,792 nucleotides were obtained for assembly (Table 1). These reads were assembled using the Trinity program, resulting in 123,433 contigs with an average length of 724 nt and an N50 of 994 nt. After clustering with CD-Hit-EST, the contigs were assembled into 69,635 transcripts with a mean size of 681 nt and N50 of 930 nt. The size distribution of the transcripts exhibited the following pattern: 25.51% (17,770) of the transcripts were less than 300 nt, 55.38% (38,564) of the transcripts ranged from 300 to 1000 nt in length, 18.09% (12,603) of the transcripts ranged from 1000 to 3000 nt, and 1.0% (698) were more than 3000 nt in length (Additional file 4: Figure S1).

**Table 1:** Summary of the transcriptome of *N. officinale*

	Raw reads	Contigs	Transcripts
Total length (bp)	5,287,387,792	89,449,846	47,428,745
Number of sequences	69,570,892	123,433	69,635
Average length (bp)	76	724	681
Median length (bp)	76	501	453
Max length (bp)	76	16,627	16,627
Min length (bp)	76	224	224
N50 (bp)	76	994	930

### Functional annotation and classification of *N. officinale* transcripts

For functional annotation, the transcripts were identified based on the BLASTX algorithm (available at the NCBI website) against the non-redundant (NR) protein database and nucleotide (NT) database with an E-value cutoff of 1 x 10^−5^ (Table 2). Of the total 69,635 transcripts, 57,550 transcripts (82.65%) had BLAST hits to known proteins in the NR database and 61,020 transcripts (87.63%) had BLAST hits to nucleotides in the NT database. In addition, some transcripts were aligned to public databases, including 46,249 (66.42%) transcripts in the SWISS-PROT protein database, 60,335 (86.64%) transcripts in the *Brassica* database (BRAD), 61,369 (88.13%) transcripts in the *Arabidopsis* information resource (TAIR) database, 16,530 (23.74%) transcripts in the Clusters of Orthologous Group (COG) database, and 45,402 (65.20%) transcripts in Gene Ontology (GO) database. In total, 64,876 transcripts were identified, representing approximately 93.17% of all assembled transcripts.

**Table 2:** Summary of annotations of the *N. officinale* transcripts

	Number of BLASTed transcripts	Ratio (%)
All transcripts	69,635	100
Transcripts BLASTed against NR	57,550	82.65
Transcripts BLASTed against NT	61,020	87.63
Transcripts BLASTed against SWISS-PROT	46,249	66.42
Transcripts BLASTed against BRAD	60,335	86.64
Transcripts BLASTed against TAIR	61,369	88.13
Transcripts BLASTed against COG	16,530	23.74
Transcripts BLASTed against GO	45,402	65.20
All annotated transcripts	64,876	93.17

*NR* NCBI non-redundant protein database, *NT* NCBI nucleotide database, *SWISS-PROT* SwissProt protein database, *BRAD Brassica rapa* protein database, *TAIR Arabidopsis* protein database, *COG* Clusters of Orthologous Group, *GO* Gene Ontology

**Fig. 2:**
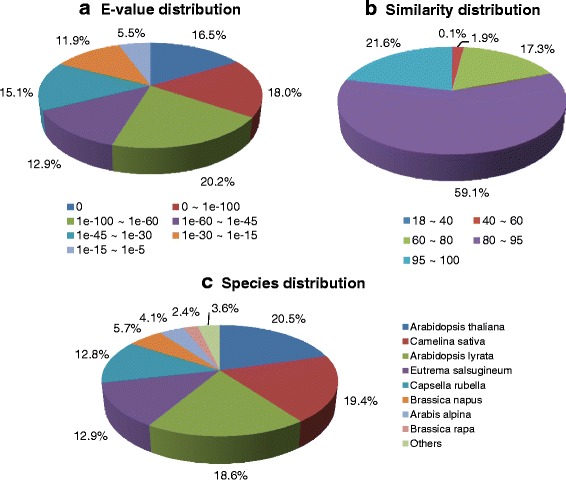
Classification of NR annotation results of the *N. officinale* transcripts. **a** E-value distribution, **b** Similarity distribution, and **c** Species distribution

The E-value distribution of the transcripts in the NR databases showed that 54.6% of aligned transcripts had strong similarity with an E-value <1e-60, whereas the remaining 45.4% of the homologous sequences ranged from 1e-5 to 1e-60 (Fig. 2a). The similarity distribution in the NR database showed that 81% of the sequences had a similarity higher than 80.7 and 19.3% of the sequences had a similarity lower than 80% (Fig. 2b). In the species distribution, the *N. officinale* transcriptome showed 20.5% similarity with that of *Arabidopsis thaliana*, with lower similarities to other species, including *Camelina sativa* (19.4%), *Arabidopsis lyrata* (18.6%), *Eutrema salsugineum* (12.9%), *Capsella rubella* (12.8%), *Brassica napus* (5.7%), *Arabis alpina* (4.1%), *Brassica rapa* (2.4%), and others (3.6%) (Fig. 2c). Most BLAST hits (approximately 96.4%) were to sequences from the Brassicaceae family. The *N. officinale* transcriptome showed highest species similarity and annotation ratio to *A. thaliana*, which is an important plant model species. *Arabidopsis* is a member of Brassicaceae family such as *N. officinale* and contains 25,498 genes encoding proteins from 11,000 families [51]. *Arabidopsis* and *N. officinale* have similar morphology and significant sequence homology, indicating the correlation between mouse-ear cress and watercress.

**Fig. 3:**
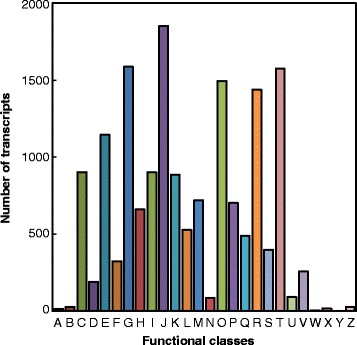
COG functional classification of the *N. officinale* transcripts. A total of 16,530 transcripts (23.74% of total) were annotated and divided into 26 subcategories. *a* RNA processing and modification; *b* Chromatin Structure and dynamics; *c* Energy production and conversion; *d* Cell cycle control, cell division, chromosome partitioning; *e* Amino acid transport and metabolism; *f* Nucleotide transport and metabolism; *g* Carbohydrate transport and metabolism; *h* Coenzyme transport and metabolism; *i* Lipid transport and metabolism; *j* Translation, ribosomal structure and biogenesis; *k* Transcription; *l* Replication, recombination and repair; *m* Cell wall/membrane/envelope biogenesis; *n* Cell motility; *o* Post-translational modification, protein turnover, chaperones; *p* Inorganic ion transport and metabolism; *q* Secondary metabolite biosynthesis, transport and catabolism; *r* General functional prediction only; *s* Function unknown; *t* Signal transduction mechanisms; *u* Intracellular trafficking, secretion, and vesicular transport; *v* Defense mechanisms; *w* Extracellular structures; *x* Phage-derived proteins, transposases and other mobilized components; *y* Nuclear structure; *z* Cytoskeleton

**Fig. 4:**
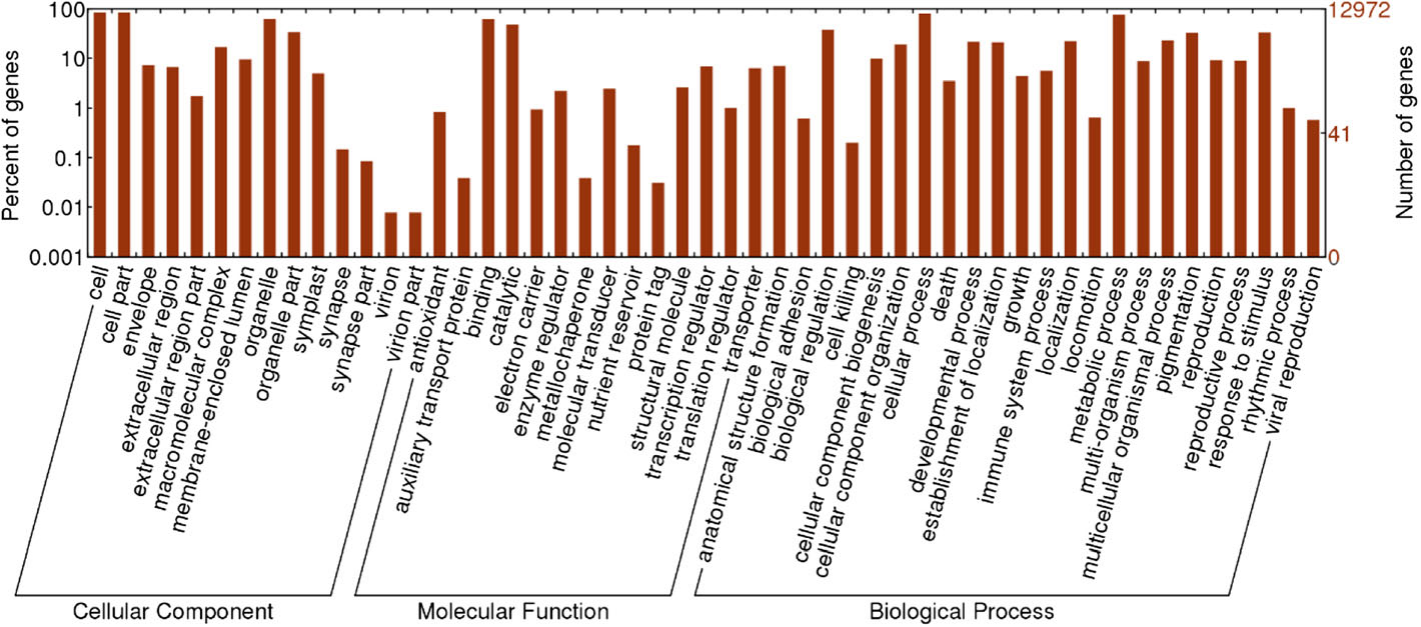
GO annotation of the *N. officinale* transcripts. A total of 45,402 transcripts (65.20% of total) were annotated and classified into three major categories (biological process, cellular component, and molecular function) and 60 subcategories

COG analysis showed that 16,530 of the total transcripts were classified into 26 molecular families (Fig. 3). Among these categories, the largest category was “translation, ribosomal structure and biogenesis” containing 1850 transcripts (11.19%), followed by “carbohydrate transport and metabolism” (1586, 9.59%), “signal transduction mechanisms” (1573, 9.51%), “post-translational modification, protein turnover, chaperones” (1494, 9.03%), and “general functional prediction only” (1437, 8.69%). “Extracellular structures” (7, 0.04%) was the smallest category, and 489 transcripts were found in the clusters of the “secondary metabolite biosynthesis, transport and catabolism” category. GO analysis revealed that 45,402 of the total assembled transcripts were distributed in 56 sub-categories under three main GO functional categories: cellular components (143,456, 34.35%), molecular function (63,191, 15.13%), and biological process (210,884, 50.50%) (Fig. 4). In the three main categories, the dominant groups of sub-categories were “cellular process” (37,328, 82.2%) and “metabolic process” (35,372, 77.9%) in the biological processes, “cell” (39,487, 87%) and “cell part” (39,436, 86.9%) in cellular components, and “binding” (29,335, 64.6%) and “catalytic” (22,882, 50.4%) in molecular functions.

### Expression analysis of glucosinolate-related genes in different organs of *N. officinale*

*Brassica rapa* has 102 putative glucosinolate genes, which are orthologs of 52 glucosinolate genes in *A. thaliana*. The homologous glucosinolate genes in *B. rapa* and *A. thaliana* share 59%-91% nucleotide sequence identity [52]. To identify the expression of genes that encode enzymes related to the glucosinolate biosynthetic pathways, we analyzed the *N. officinale* transcriptome dataset. On the basis of the functional annotation of the *N. officinale* transcriptome, we found that seven glucosinolate transcription factors and 26 glucosinolate biosynthetic genes were highly similar to those of species belonging to the Brassicaceae such as *A. thaliana*, *B. oleracea*, and *B. rapa* (Table 3). *NoMYB28* was shown to have 83% similarity with *A. thaliana MYB28* (NP_200950.1), 77% similarity with *B. rapa MYB28* (ADQ92843.1), and 74% similarity with *B. oleracea MYB28* (CBI71385.1). Similarly, other *N. officinale* glucosinolate biosynthetic genes showed more than 67% similarity with other orthologous genes. By comparing other orthologous genes, we identified several full-length cDNA clones encoding *MYB28*, *MYB29*, *BCAT4*, *MAM1*, *CYP79F1*, *CYP83A1*, *GGP1*, *SUR1*, *UGT74B1*, *ST5b*, *ST5a*, *ST5c*, *FMO GS-OX5*, *CYP79B2*, *GSTF9*, and *IGMT*, and partial-length cDNA clones encoding *MYB34*, *MYB51*, *MYB122*, *IQD1-1*, *Dof1.1*, *MAM3*, *GSTF11*, *GSTF20*, *UGT74C1*, *FMO GS-OX2*, *CYP79B3*, *CYP83B1*, *GSTF10*, *CYP81F2*, *CYP81F3*, *PEN2*, and *TGG2*.

**Table 3:** Comparison of glucosinolate-related genes of *N.officinale* with the most orthologous genes

Genes	Length (amino acid)	Sequence form	Orthologous genes (Accession no.)	Identity (%)
*NoMYB28*	370	Full-length	*Arabidopsis thaliana MYB28* (NP_200950.1)	83
			*Brassica rapa MYB28* (ADQ92843.1)	77
			*Brassica oleracea MYB28* (CBI71385.1)	74
*NoMYB29*	350	Full-length	*Arabidopsis thaliana MYB29* (NP_196386.13)	78
			*Brassica juncea MYB29-2* (AFY09821.1)	75
			*Brassica oleracea MYB29* (AKD49017.1)	71
*NoMYB34*	53	Partial-length	*Brassica oleracea MYB34* (BAM78216.1)	98
			*Brassica rapa MYB34-3* (ADV17461.1)	98
			*Arabidopsis thaliana MYB34* (NP_200897.1)	96
*NoMYB51*	122	Partial-length	*Eruca vesicaria MYB51* (AGS49160.1)	81
			*Arabidopsis thaliana MYB51* (NP_173292.1)	90
			*Brassica rapa MYB51-1* (ACR48187.1)	80
*NoMYB122*	35	Partial-length	*Arabidopsis thaliana MYB122* (NP_177548.1)	94
			*Arabidopsis lyrata MYB122* (XP_002887524.1)	94
			*Brassica rapa MYB122* (XP_009106064.1)	91
*NoIQD1-1*	81	Partial-length	*Arabidopsis thaliana IQD1* (NP_187582.1)	86
			*Camelina sativa IQD1* (XP_010464624.1)	81
			*Brassica rapa IQD1* (XP_009123236.1)	76
*NoDof1.1*	256	Partial-length	*Arabidopsis thaliana Dof 1.1*(NP_850938.1)	78
			*Camelina sativa Dof1.1* (XP_010487779.1)	79
			*Brassica rapa Dof1.1* (XP_009110928.1)	68
*NoBCAT4*	352	Full-length	*Brassica oleracea BCAT4* (AJF21970.1)	83
			*Brassica rapa BCAT4* (ACR10245.1)	82
			*Arabidopsis lyrata BCAT4* (XP_002885325.1)	79
*NoMAM1*	500	Full-length	*Arabidopsis thaliana MAM1* (NP_197692.1)	81
			*Camelina sativa MAM1* (XP_010454580.1)	80
			*Brassica rapa MAM1* (XP_009130133.1)	75
*NoMAM3*	302	Partial-length	*Camelina sativa MAM3* (XP_010428949.1)	82
			*Boechera divaricarpa MAM3* (CAJ55514.1)	82
			*Arabidopsis thaliana MAM3* (NP_197693.1)	81
*NoCYP79F1*	539	Full-length	*Brassica oleracea CYP79F1* (ACB59213.1)	84
			*Brassica napus CYP79F1* (AGO59948.1)	83
			*Brassica rapa CYP79F1* (ACR10252.1)	83
*NoCYP83A1*	502	Full-length	*Arabidopsis thaliana* CYP83A1 (NP_193113.1)	88
			*Raphanus sativus CYP83A1* (AHB11194.1)	88
			*Brassica oleracea CYP83A1* (AIK28472.1)	87
*NoGSTF11*	214	Partial-length	*Arabidopsis lyrata GSTF11* (XP_002882279.1)	91
			*Camelina sativa GSTF11* (XP_010463766.1)	91
			*Arabidopsis thaliana GSTF11* (NP_186969.1)	88
*NoGSTF20*	94	Partial-length	*Arabidopsis thaliana GSTF20* (NP_177958.1)	91
			*Camelina sativa GSTF20* (XP_010472052.1)	91
			*Brassica oleracea GSTF20* (XP_013592747.1)	88
*NoGGP1*	250	Full-length	*Brassica rapa GGP1* (XP_009108982.1)	87
			*Brassica oleracea GGP1* (XP_013598031.1)	88
			*Camelina sativa GGP1* (XP_010438166.1)	87
*NoSUR1*	376	Full-length	*Arabidopsis thaliana SUR1* (NP_179650.1)	90
			*Eruca vesicaria SUR1* (AGS49169.1)	89
			*Brassica rapa SUR1* (ACH41755.1)	89
*NoUGT74B1*	459	Full-length	*Arabidopsis thaliana UGT74B1* (XP_010477734)	88
			*Camelina sativa UGT74B1* (XP_010477734)	85
			*Brassica rapa UGT74B1* (XP_009115475.1)	84
*NoUGT74C1*	237	Partial-length	*Arabidopsis thaliana UGT74C1* (NP_180738.1)	89
			*Camelina sativa UGT74C1* (XP_010469678.1)	89
			*Brassica oleracea UGT74C1* (XP_013637126.1)	87
*NoST5b*	341	Full-length	*Arabidopsis thaliana ST5b* (NP_177549.1)	84
			*Camelina sativa ST5b* (XP_010416243.1)	80
			*Brassica rapa ST5b* (XP_009106065.1)	80
*NoST5a*	337	Full-length	*Arabidopsis thaliana ST5a* (NP_177550.1)	96
			*Brassica rapa ST5a* (ACR10265.1)	91
			*Camelina sativa ST5a* (XP_010419185.1)	96
*NoST5c*	350	Full-length	*Arabidopsis thaliana ST5c* (NP_173294.1)	89
			*Camelina sativa ST5c* (XP_010459484.1)	87
			*Brassica rapa ST5c* (ACR10273.1)	85
*NoFMO* *GS-OX2*	122	Partial-length	*Camelina sativa FMO GS-OX2* (XP_010473477.1)	85
			*Brassica oleracea FMO GS-OX2* (XP_013612296.1)	80
			*Brassica rapa FMO GS-OX2* (XP_009113068.1)	79
*NoFMO* *GS-OX5*	456	Full-length	*Arabidopsis thaliana FMO GS-OX5* (NP_172678.3)	86
			*Brassica oleracea FMO GS-OX5* (FMO GS-OX5)	86
			*Brassica rapa FMO GS-OX5* (XP_009110664.1)	85
*NoCYP79B2*	541	Full-length	*Arabidopsis lyrata CYP79B2* (XP_002866896.1)	94
			*Brassica oleracea CYP79B2* (ADW54459.1)	94
			*Eruca vesicaria CYP79B2* (AGM16417.1)	93
*NoCYP79B3*	144	Partial-length	*Brassica rapa CYP79B3* (ACR10255.1)	91
			*Arabidopsis lyrata CYP79B3* (XP_002878610)	90
			*Brassica napus CYP79B3* (AAN76810.1)	73
*NoCYP83B1*	448	Partial-length	*Brassica oleracea CYP83B1* (ADW54460.1)	96
			*Arabidopsis thaliana CYP83B1* (NP_194878.1)	95
			*Raphanus sativus CYP83B1* (AHB11193.1)	96
*NoGSTF9*	213	Full-length	*Brassica rapa GSTF9* (XP_009132756.1)	97
			*Brassica oleracea GSTF9* (XP_013636508.1)	97
			*Arabidopsis thaliana GSTF9* (NP_180643.1)	96
*NoGSTF10*	48	Partial-length	*Brassica rapa GSTF10* (XP_009132757.1)	90
			*Brassica oleracea GSTF10* (XP_013622385.1)	90
			*Arabidopsis thaliana GSTF10* (NP_180644.1)	88
*NoCYP81F2*	170	Partial-length	*Arabidopsis thaliana CYP81F2* (NP_200532.1)	89
			*Arabidopsis lyrata CYP81F2* (XP_002864506.1)	89
			*Arabis alpina CYP81F2* (AEM44335.1)	81
*NoCYP81F3*	231	Partial-length	*Arabidopsis thaliana CYP81F3* (NP_568025.1)	93
			*Arabidopsis lyrata CYP81F3* (XP_002868991.1)	93
			*Brassica napus CYP81F3* (CDY44041.1)	92
*NoIGMT*	373	Full-length	*Arabidopsis thaliana IGMT* (NP_173534.1)	91
			*Camelina sativa IGMT* (XP_010498564.1)	90
			*Brassica rapa IGMT* (XP_009149591.1)	91
*NoPEN2*	405	Partial-length	*Arabidopsis thaliana PEN2* (NP_181977.1)	90
			*Arabis alpina PEN2* (AEM44334.1)	90
			*Brassica rapa PEN2* (XP_009143040.1)	87
*NoTGG2*	532	Partial-length	*Armoracia rusticana TGG2* (AAV71147.1)	90
			*Arabidopsis thaliana TGG2* (BAE98479.1)	70
			*Tarenaya hassleriana TGG2* (XP_010519862.1)	67

**Fig. 5:**
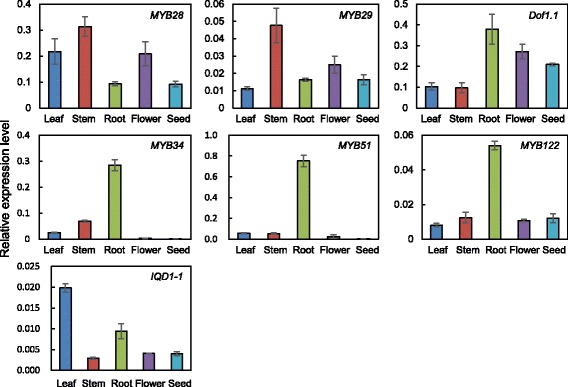
Expression of glucosinolate transcription factors in different organs of *N. officinale*. The expression level was measured in 2-month-old *N. officinale*. Relative expression level was plotted after normalization to *UBC9*. Mean values and SDs from triplicate biological experiments are plotted

**Fig. 6:**
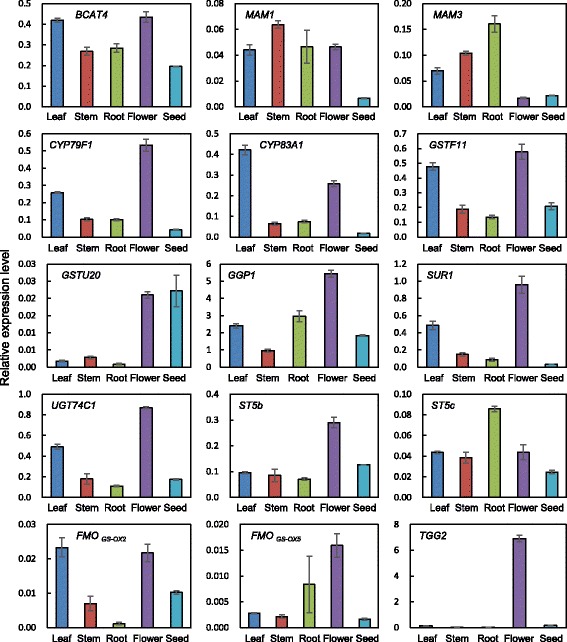
Expression of aliphatic glucosinolate biosynthetic genes in different organs of *N. officinale*. The expression level was measured in 2-month-old *N. officinale*. Relative expression level was plotted after normalization to *UBC9*. Mean values and SDs from triplicate biological experiments are plotted

**Fig. 7:**
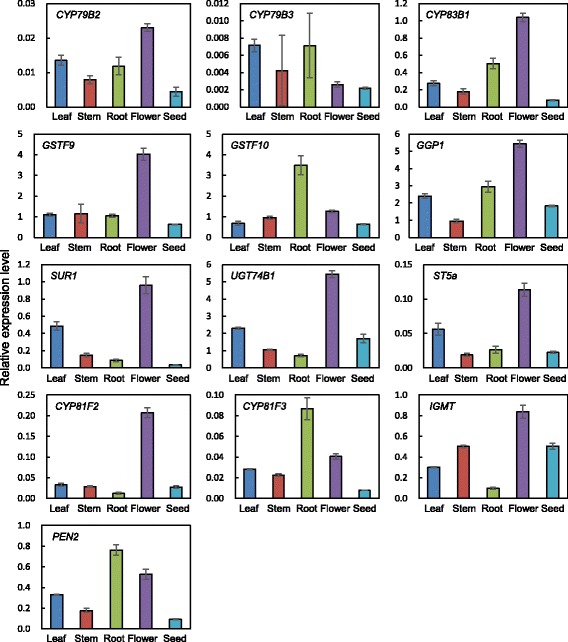
Expression of indolic glucosinolate biosynthetic genes in different organs of *N. officinale*. The expression level was measured in 2-month-old *N. officinale*. Relative expression level was plotted after normalization to *UBC9*. Mean values and SDs from triplicate biological experiments are plotted

The expression of glucosinolate-related transcription factors was analyzed in the leaves, stems, roots, flowers, and seeds of *N. officinale* by quantitative RT-PCR (Fig. 5). The expression of *NoMYB28* and *NoMYB29* was highest in the stems, which is consistent with the transcript levels of *BrMYB28, BrMYB29-2,* and *BrMYB29-3* in the stems of *B. rapa* [53]. *NoMYB34, NoMYB51, NoMYB122*, and *NoDof1.1* were more strongly expressed in the roots compared with other organs. Finally, the highest expression of *NoIQD1-1*, which is involved in both aliphatic and indolic glucosinolate biosynthesis, was observed in leaves. Most glucosinolate biosynthetic genes were more highly expressed in the flowers compared with the leaves, stems, roots, and seeds. However, *NoMAM1*, *NoMAM3*, *NoCYP83A1*, *NoGSTU20*, *NoST5c*, and *NoFMO GS-OX2*, which are involved in aliphatic glucosinolate biosynthesis, had the highest expression levels in stems, roots, leaves, seeds, roots, and leaves, respectively (Fig. 6). In addition, among the indolic glucosinolate biosynthetic genes, the highest expression levels of *NoCYP79B3, NoGSTF10, NoCYP81F3,* and *NoPEN2* were observed in roots (Fig. 7).

### Analysis of glucosinolate content in different organs of *N. officinale*

In HPLC analysis, we identified eight different glucosinolates in the different organs of *N. officinale*; glucoiberin, glucotropaeolin, 4-hydroxyglucobrassicin, glucosiberin, glucohirsutin, glucobrassicin, 4-methoxyglucobrassicin, and gluconasturtiin (Table 4). The levels of these glucosinolates distributed over the different organs of *N. officinale* (Table 5). The amount of total glucosinolates was highest in the flower, 6.1, 3.0, 2.3, and 1.2 times higher than that in the root, stem, leaf, and seed, respectively. Among the eight glucosinolates, the level of gluconasturtiin was considerably higher than any other glucosinolate, irrespective of the organ. In particular, the gluconasturtiin content in the flower and seed was considerably higher than that in other organs. The content of gluconasturtiin in the flower was 9.8, 2.9, 2.2, and 1.3 times higher than that in the root, stem, leaf, and seed, respectively. The content of glucotropaeolin was also highest in the flower, with concentrations 12.0, 4.5, and 2.3 times higher than that in the stem, leaf, and root, respectively. The second highest level of total glucosinolates was observed in the seed. The seed contains higher amounts of glucoiberin, glucosiberin, and glucohirsutin than the other organs of *N. officinale*. The level of glucoiberin was 7.9, 5.6, 3.3, and 1.8 times higher in the seed than in the root, leaf, stem, and flower, respectively. The content of glucosiberin was highest in the seed, with levels 8.6, 7.8, 6.8, and 1.4 times higher than those in the stem, root, leaf, and flower, respectively. The amount of glucohirsutin was highest in the seed, being 9.6, 7.6, 6.5, and 1.6 times higher than that in the stem, leaf, root, and flower, respectively. Although the total glucosinolate content was lowest in the root, the amount of 4-hydroxyglucobrassicin was highest in the root, with levels 36.8, 32.7, 26.7, and 2.9 times higher than that in the seed, stem, leaf, and flower, respectively. The root also contained the highest amount of glucobrassicin.

**Table 4:** Glucosinolates identified by LC-ESI/MS in *N. officinale*

Classification	Trivial name	Chemical formula	R side chain	Molecular weight^a^
Aliphatic	Glucoiberin	CH_3_SO(CH_2_)_3_	3-(methylsulfinyl)propyl	343.18
	Glucosiberin	CH_3_SO(CH_2_)_7_	7-(methylsulfinyl)heptyl	399.29
	Glucohirsutin	CH_3_SO(CH_2_)_8_	8-(methylsulfinyl)octyl	413.32
Indole	Glucobrassicin	C_8_H_6_NCH_2_	3-indolylmethyl	368.17
	4-Hydroxyglucobrassicin	4-OHC_8_H_6_NCH_2_	4-hydroxy-3-indolylmethyl	384.17
	4-Methoxyglucobrassicin	4-(CH_3_O)C_8_H_6_NCH_2_	4-methoxy-3-indolylmethyl	398.20
Aromatic	Gluconasturtiin	C_6_H_5_(CH_2_)_2_	2-phenylethyl	343.16
	Glucotropaeolin	C_6_H_5_CH_2_	Benzyl	329.13

Molecular weight^a^: molecular weight of desulfo-glucosinolates

**Table 5:** Glucosinolate contents in different organs of *N. officinale*

Glucosinolates	Leaf	Stem	Root	Flower	Seed
Glucoiberin	0.14 ± 0.01	0.24 ± 0.02	0.10 ± 0.02	0.43 ± 0.01	0.79 ± 0.75
Glucotropaeolin	0.08 ± 0.04	0.03 ± 0.03	0.16 ± 0.02	0.36 ± 0.05	0.33 ± 0.10
4-Hydroxyglucobrassicin	0.11 ± 0.02	0.09 ± 0.00	2.94 ± 0.17	1.02 ± 1.56	0.08 ± 0.03
Glucosiberin	1.00 ± 0.03	0.79 ± 0.01	0.86 ± 0.10	4.91 ± 3.64	6.75 ± 1.58
Glucohirsutin	0.42 ± 0.00	0.33 ± 0.01	0.49 ± 0.05	2.01 ± 1.39	3.18 ± 0.79
Glucobrassicin	0.40 ± 0.02	0.53 ± 0.03	0.96 ± 0.06	0.75 ± 0.02	0.13 ± 0.03
4-Methoxyglucobrassicin	0.19 ± 0.00	0.78 ± 0.04	0.69 ± 0.03	0.17 ± 0.01	0.13 ± 0.01
Gluconasturtiin	33.77 ± 0.73	25.20 ± 0.72	7.56 ± 0.26	73.90 ± 0.93	56.68 ± 7.45
Total	36.13 ± 0.84	27.98 ± 0.86	13.76 ± 0.71	83.55 ± 7.61	68.07 ± 10.73

Total glucosinolates were measured in 2-month-old *N. officinale* (μg g^−1^ dry weight). Each value represents the mean of three replicates and error bars are SDs

## Discussion

Despite the health-benefiting importance and economical value of watercress, there is still limited genomic and physiological information available for *N. officinale*. In this study, we performed comparative analyses of the phytonutritional property of *N. officinale*, using both transcriptomic and metabolomics approaches. In our transcriptome analysis of *N. officinale* seedlings, we revealed total 69,635 transcripts and annotated 64,876 (93.17%) of total transcripts of *N. officinale* using public databases. On the basis of the annotations and sequence identities of *N. officinale*, we identified 33 candidate genes encoding enzymes related to glucosinolate biosynthetic pathways and analyzed the expression of these genes in the leaves, stems, roots, flowers, and seeds of *N. officinale*. Furthermore, we also profiled glucosinolate metabolic data via HPLC-UV analysis and identified eight glucosinolates in different organs of *N. officinale*. Among these eight glucosinolates, the level of gluconasturtiin was considerably higher than any other glucosinolate in individual organs. These transcriptomic and metabolomics results are highly consistent with those obtained in a recently published study by Voutsina et al. [8]. These authors performed RNA-sequencing analysis of 12 watercress accessions to investigate the genetic basis of two key watercress nutritional attributes: antioxidant (AO) capacity and glucosinolate (GLS) content. The transcriptome analysis of *N. officinale* yielded 80,800 transcripts (48,732 unigenes), of which 54,595 (67.6%) transcripts were annotated using a BLASTx search against *Arabidopsis*. Differentially expressed gene (DEG) analysis comparing watercress accessions with “high” and “low” AO and GLS revealed 145 and 94 differentially expressed loci for AO capacity and GLS, respectively. DEG analysis between the high and low GLS watercress identified links to GLS regulation and novel transcripts warranting further investigation. In the DEG analysis, they identified two differentially expressed shikimate pathway genes, c33663_g1_i2; similar to shikimate kinases and c37926_g1_i6; dehydroquinateshikimate dehydrogenase, acting upstream of the glucosinolate pathway. Our transcriptome data for *N. officinale* also revealed seven putative genes encoding glucosinolate transcription factors and 26 putative glucosinolate biosynthetic genes (Table 3). The seven putative genes encoding glucosinolate transcription factors, *NoMYB28, NoMYB29, NOMYB34, NoMYB51, NoMYB122, NoDof1.1,* and *NoIQD1.1*, are thought to act in glucosinolate biosynthesis regulation [27–31]. Intensive research on the relationship between shikimate pathway genes and glucosinolate biosynthetic genes in watercress will enhance our understanding of functional genomic approach, including glucosinolate biosynthetic pathways.

Many indole glucosinolate biosynthetic genes are specifically expressed at highest levels in the roots and flowers. In *N. officinale*, the accumulation patterns of indole glucosinolates, such as 4-hydroxyglucobrassicin and glucobrassicin, coincide with the expression patterns of the genes related to these indole glucosinolates. In contrast, the accumulation patterns of aliphatic glucosinolates did not coincide with the expression pattern of aliphatic glucosinolate-related genes in *N. officinale*. Most of the aliphatic glucosinolate biosynthetic genes were more highly expressed in the flowers compared with the leaves, stems, roots, and seeds, whereas the contents of aliphatic glucosinolates, such as glucoiberin, glucosiberin, and glucohirsutin, were relatively higher in the seed. Several genes involved in the regulation of glucosinolate biosynthetic pathways and external stimulations could be linked to accumulate the glucosinolate contents [54, 55]. Although there are many reasons for this discordance, the shift in developmental stage from flower to seed might possibly explain the discrepancy between gene expression pattern and metabolite content.

## Conclusions

In RNA sequencing analysis using an Illumina NextSeq500 sequencer, we identified a total 69,635 transcripts and annotated 64,876 transcripts, which provide basic information for further research on the secondary metabolites in *N. officinale*. Our transcriptome data reveal that several genes encoding enzymes related to glucosinolate biosynthetic pathways are well conserved in *N. officinale* and that these genes have high similarity to those in other cruciferous plants such as *Arabidopsis thaliana*, *Brassica rapa*, and *Camelina sativa*.

On the basis of our gene expression study and HPLC analysis, we identified that most glucosinolate biosynthetic genes are highly expressed in flowers and that the content of total glucosinolates was also higher in flowers than in other organs, indicating a positive correlation between the expression of glucosinolate-related genes and glucosinolate contents in different organs of *N. officinale*. The results of this research provide comprehensive information on the *N. officinale* genome and enhance our understanding of the glucosinolate biosynthesis pathways in this plant.

## Additional files

Additional file 1: EXCEL file including detailed analysis of transcripts in *Nasturtium officinale*. (XLSX 15290 kb)

Additional file 2: EXCEL file including sequence of transcripts in *Nasturtium officinale*. (XLSX 36722 kb)

Additional file 3: Table S1. Primers used in this work. (DOCX 17.9 kb)

Additional file 4: Figure S1. Length distribution of contigs and transcripts in *N. officinale*. (PPTX 53.1 kb)

## Abbreviations

ATR1: Altered tryptophan regulation1
BAT: Bile acid transporter
BCAT4: Branched-chain aminotransferase4
CYP83A1: Cytochrome P450 family 83 subfamily A polypeptide 1
FMOGS-OX5: Flavin-monooxygenase glucosinolate S-oxygenase5
GGP1: Class I glutamine amidotransferase-like superfamily protein
GSTF9: Glutathione S-transferase PHI9
HAG1: High aliphatic glucosinolate1
HIG1: High indolic glucosinolate1
I3C: Indole-3-carbinol
MAM1: Methylthioalkylmalate synthase1
ST5B: Sulfotransferase5b
SUR1: Tyrosine transaminase family protein
UGT74B1: UDP-glucosyl transferase74B1
