# Supplementary material for: De novo transcriptome analysis and glucosinolate profiling in watercress (Nasturtium officinale R. Br.)
Source: BMC Genomics. 2017 May 23;18:401. doi: 10.1186/s12864-017-3792-5 (PMC5442658; doi:10.1186/s12864-017-3792-5)
Supplement: Supplementary file 3 — Primers used in this work. (DOCX 17.9 kb) [file 12864_2017_3792_MOESM3_ESM.docx]

**Table S1** Primers used in this work

| **Primer** | **Sequence (5' to 3')** | **Amplication (bp)** |
| --- | --- | --- |
| *NoUBC9* F | GACCCGTCGCGGAAGACAT | 189 |
| *NoUBC9* R | CGAGACAGATGCTCCCATTGC |  |
| *NoMYB28* F | TCCCACGACTCACAAGCCACT | 171 |
| *NoMYB28* R | ATCTCGAACACCTCGGAAGTCG |  |
| *NoMYB29* F | CCGTGACCCACAAGCCACTT | 163 |
| *NoMYB29* R | TCACCGCTGCTTATCTCCGGTA |  |
| *NoMYB34* F | TCTTCCCACATCTCTTCAACCCA | 154 |
| *NoMYB34* R | ATGGTGAGGACGCCATGCTG |  |
| *NoMYB51* F | CCCTTCACGGCAACAAATGG | 161 |
| *NoMYB51* R | TGATTTGTCGGTGCCCGAGT |  |
| *NoMYB122* F | GCACACCATGTTGTAGAGCAGAGG | 97 |
| *NoMYB122* R | CCTTCACCATGCCGTTGAACA |  |
| *NoIQD1-1* F | GGATCCTAGCAACCCGACTTGG | 165 |
| *NoIQD1-1* R | CCTGGATTCTCCCATGGCCG |  |
| *NoDOF1.1* F | GGGCTCTACGCTTTACCGAACG | 164 |
| *NoDOF1.1* R | GAAGTCAAACCGGCGACTGG |  |
| *NoBCAT4* F | CTGGGACTGCTGCAATCGTG | 159 |
| *NoBCAT4* R | CCACCGTCCATCCCTTCGTA |  |
| *NoMAM1* F | GCCATTGCACGATGCAAACC | 156 |
| *NoMAM1* R | CACGGCCATCTCGATCACTTC |  |
| *NoMAM3* F | CGGCAGGACGGAGAAGGACT | 173 |
| *NoMAM3* R | TGGCGAGGACAACATCCTCA |  |
| *NoCYP79F1* F | GCGGACCGGCCTCATCTT | 177 |
| *NoCYP79F1* R | GAGGTTGTCCGCTTCGATGG |  |
| *NoCYP83A1* F | CGGTGATCGGAAACCTCCAC | 182 |
| *NoCYP83A1* R | CCGGTCCGCAAAGTTGACAT |  |
| *NoGSTF11* F | GCGGACCAAGGAACGGATCT | 172 |
| *NoGSTF11* R | GCTCCTCGACCAAAGCGATG |  |
| *NoGSTF20* F | CCGGTCCTGATCCACAATGG | 151 |
| *NoGSTF20* R | TCTTGTCTACGAAATCAGCCCAGAA |  |
| *NoGGP1* F | TGGTGTTGCCCGAATCTGCT | 181 |
| *NoGGP1* R | GCATCCGCAAATTCTTGCTTG |  |
| *NoSUR1* F | GTCCCGGAGCTGGGATTCTC | 152 |
| *NoSUR1* R | GTTTGGTCGAGCCAATGCGT |  |
| *NoUGT74B1* F | CGTTTGTGGGACGTCATTGGT | 174 |
| *NoUGT74B1* R | TGGGTCCGATCAACGTAGCCT |  |
| *NoUGT74C1* F | ATGTGGCGTTCGGGACATTG | 172 |
| *NoUGT74C1* R | GCCACAAGTCCACAGCCTTTCT |  |
| *NoST5b* F | TGACGACTCCTCGAACCCTCTC | 165 |
| *NoST5b* R | TTCGCAACCGACTCCGGTAA |  |
| *NoST5a* F | TTCACGCCAAAGACCACTTCG | 150 |
| *NoST5a* R | TACGTTTGAGGAGTGGGTTCGTG |  |
| *NoST5c* F | CGTTCGTCGAGTACGGTGGTC | 177 |
| *NoST5c* R | CTTCGAATTCTGAACGGTTCGC |  |
| *NoFMO GS-OX 1* F | CGTGAAGGTCACACCGTCGTAG | 200 |
| *NoFMO GS-OX 1* R | CCACGTGGCACGAAAGGATAGT |  |
| *NoFMO GS-OX 5* F | CGCCAAGATGGATCGGTTGT | 175 |
| *NoFMO GS-OX 5* R | GAGCAAGTGCGGGTGGAAAC |  |
| *NoCYP79B2* F | CCGATCCTCACGGGACTTGA | 200 |
| *NoCYP79B2* R | TCGGCAGTAAGCAATGGGTTG |  |
| *NoCYP79B3* F | TTTCAGATGGCTCCACAGCCTTA | 151 |
| *NoCYP79B3* R | GAGTGGTCTTGACGCGAAGAGTG |  |
| *NoCYP83B1* F | CGCAGACGCAAAGATTGGTG | 192 |
| *NoCYP83B1* R | CCCGACCCGAAAGGTAGGAG |  |
| *NoGSTF9* F | CACCGTTCCTGCTGTTGTCG | 158 |
| *NoGSTF9* R | TTGCTTCAACGTCGAGCCATT |  |
| *NoGSTF10* F | TGCGCCTTTATTCGCTTCTTCA | 122 |
| *NoGSTF10* R | TCGCGATGTACTCAGGATTCCTCT |  |
| *NoCYP81F2* F | ATAGGAACCGCCGCTTACGG | 170 |
| *NoCYP81F2* R | TCAAGCTCAACGACACGACCA |  |
| *NoCYP81F3* F | CGAGCTCGAGCCACTCTTGTCT | 160 |
| *NoCYP81F3* R | GGGATGTCTCGCGCCACTAC |  |
| *NoIGMT* F | GATGTCCCAACCGGAGATGC | 159 |
| *NoIGMT* R | CCCATTCTCTGCGTTGTCAGG |  |
| *NoPEN2* F | ACATTGAACGAGCCGTGGGT | 158 |
| *NoPEN2* R | TCTGCGTGCGCAATAAGCAT |  |
| *NoTGG2* F | TGCAACGGGAACAGATGCACC | 194 |
| *NoTGG2* R | TCGCCTGCGTAACAACCTTTG |  |
