# Supplementary material for: De novo transcriptome analysis and glucosinolate profiling in watercress (Nasturtium officinale R. Br.)
Source: BMC Genomics. 2017 May 23;18:401. doi: 10.1186/s12864-017-3792-5 (PMC5442658; doi:10.1186/s12864-017-3792-5)
Supplement: Supplementary file 4 — Length distribution of contigs and transcripts in N. officinale. (PPTX 53.1 kb) [file 12864_2017_3792_MOESM4_ESM.pptx]

## Slide 1
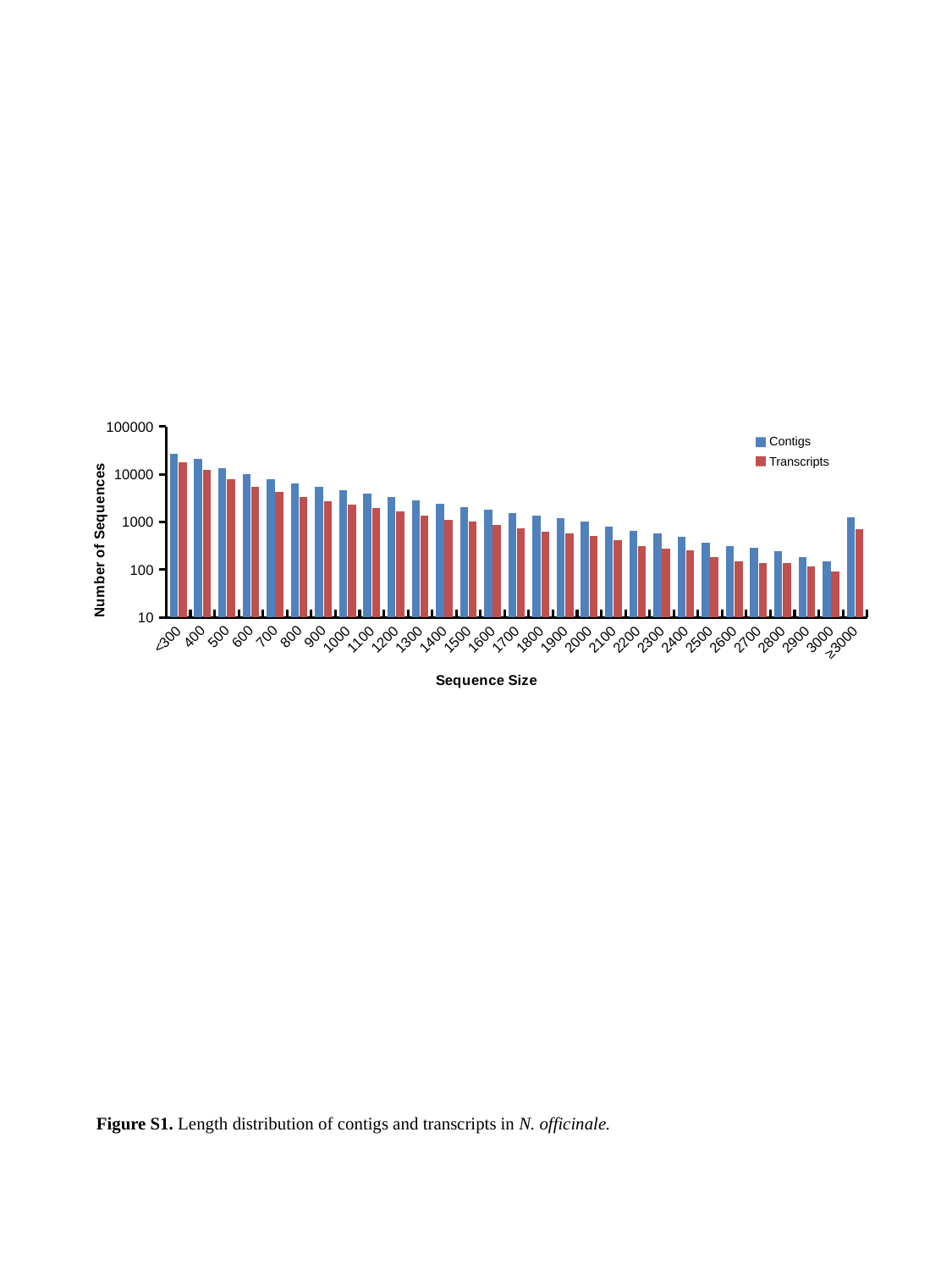

### Chart
| Category | Contigs | Unigenes |
|---|---|---|
| <300 | 27221.0 | 17770.0 |
| 400 | 20776.0 | 12553.0 |
| 500 | 13657.0 | 7850.0 |
| 600 | 10254.0 | 5576.0 |
| 700 | 7973.0 | 4260.0 |
| 800 | 6453.0 | 3329.0 |
| 900 | 5388.0 | 2710.0 |
| 1000 | 4672.0 | 2286.0 |
| 1100 | 4012.0 | 1952.0 |
| 1200 | 3412.0 | 1677.0 |
| 1300 | 2853.0 | 1392.0 |
| 1400 | 2448.0 | 1134.0 |
| 1500 | 2063.0 | 1027.0 |
| 1600 | 1807.0 | 877.0 |
| 1700 | 1530.0 | 740.0 |
| 1800 | 1380.0 | 641.0 |
| 1900 | 1196.0 | 580.0 |
| 2000 | 1027.0 | 502.0 |
| 2100 | 790.0 | 416.0 |
| 2200 | 646.0 | 308.0 |
| 2300 | 582.0 | 282.0 |
| 2400 | 485.0 | 251.0 |
| 2500 | 369.0 | 187.0 |
| 2600 | 308.0 | 153.0 |
| 2700 | 291.0 | 140.0 |
| 2800 | 241.0 | 136.0 |
| 2900 | 188.0 | 117.0 |
| 3000 | 148.0 | 91.0 |
| ≥3000 | 1263.0 | 698.0 |Contigs
Transcripts
Figure S1. Length distribution of contigs and transcripts in N. officinale.
